# Supplementary material for: Human Cytomegalovirus miR-UL70-3p Downregulates the H2O2-Induced Apoptosis by Targeting the Modulator of Apoptosis-1 (MOAP1)
Source: Int J Mol Sci. 2021 Dec 21;23(1):18. doi: 10.3390/ijms23010018 (PMC8744590; doi:10.3390/ijms23010018)
Supplement: Supplementary file 1 [file ijms-23-00018-s001.zip › Figure S1.pptm]

## Slide 1
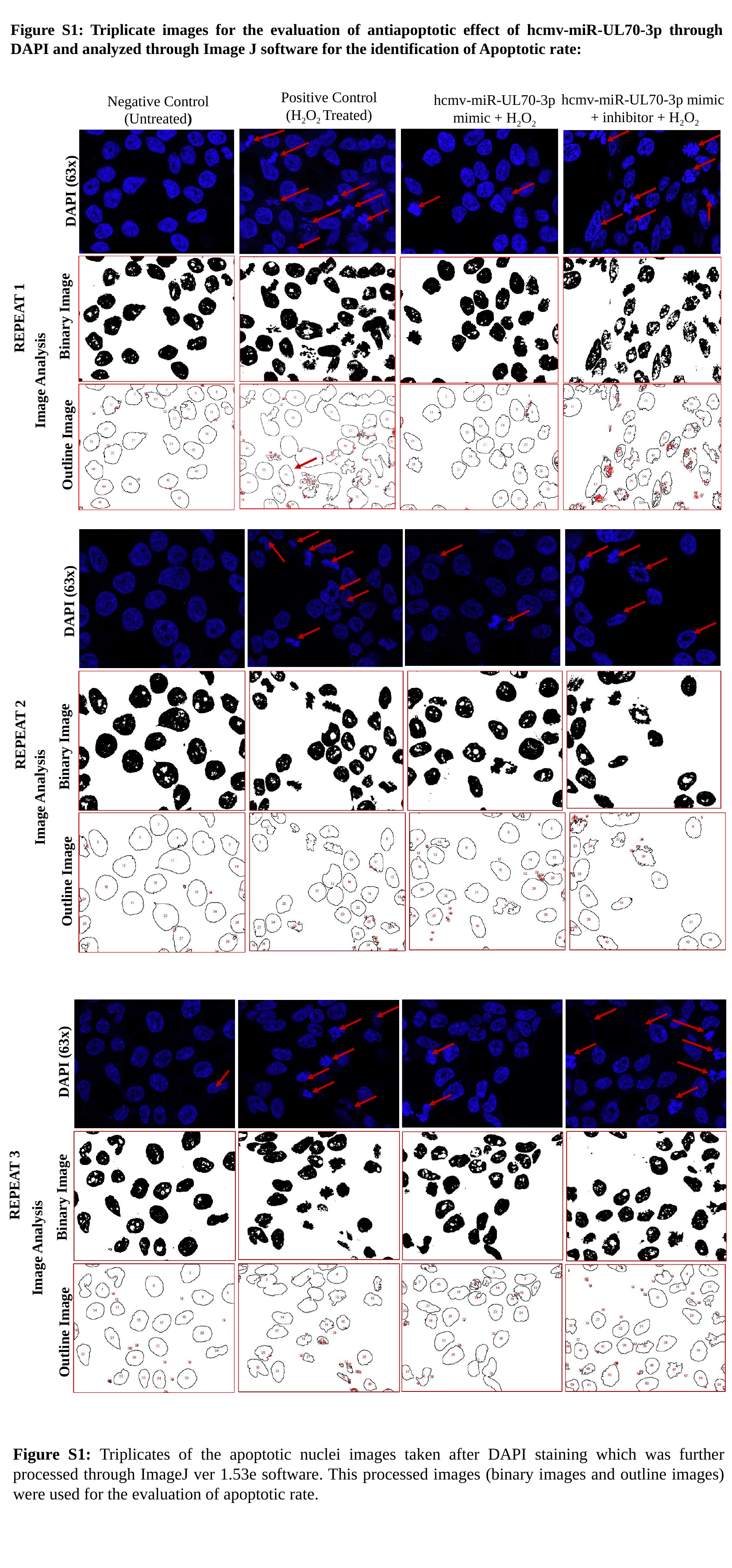

Figure S1: Triplicate images for the evaluation of antiapoptotic effect of hcmv-miR-UL70-3p through DAPI and analyzed through Image J software for the identification of Apoptotic rate:
Positive Control (H2O2 Treated)
hcmv-miR-UL70-3p mimic
+ inhibitor + H2O2
hcmv-miR-UL70-3p mimic + H2O2
Negative Control (Untreated)
DAPI (63x)
Binary Image
REPEAT 1
Image Analysis
Outline Image
DAPI (63x)
REPEAT 2
Binary Image
Image Analysis
Outline Image
DAPI (63x)
REPEAT 3
Binary Image
Image Analysis
Outline Image
Figure S1: Triplicates of the apoptotic nuclei images taken after DAPI staining which was further processed through ImageJ ver 1.53e software. This processed images (binary images and outline images) were used for the evaluation of apoptotic rate.
