# Supplementary material for: Human Cytomegalovirus miR-UL70-3p Downregulates the H2O2-Induced Apoptosis by Targeting the Modulator of Apoptosis-1 (MOAP1)
Source: Int J Mol Sci. 2021 Dec 21;23(1):18. doi: 10.3390/ijms23010018 (PMC8744590; doi:10.3390/ijms23010018)
Supplement: Supplementary file 1 [file ijms-23-00018-s001.zip › Figure S2.pptx]

## Slide 1
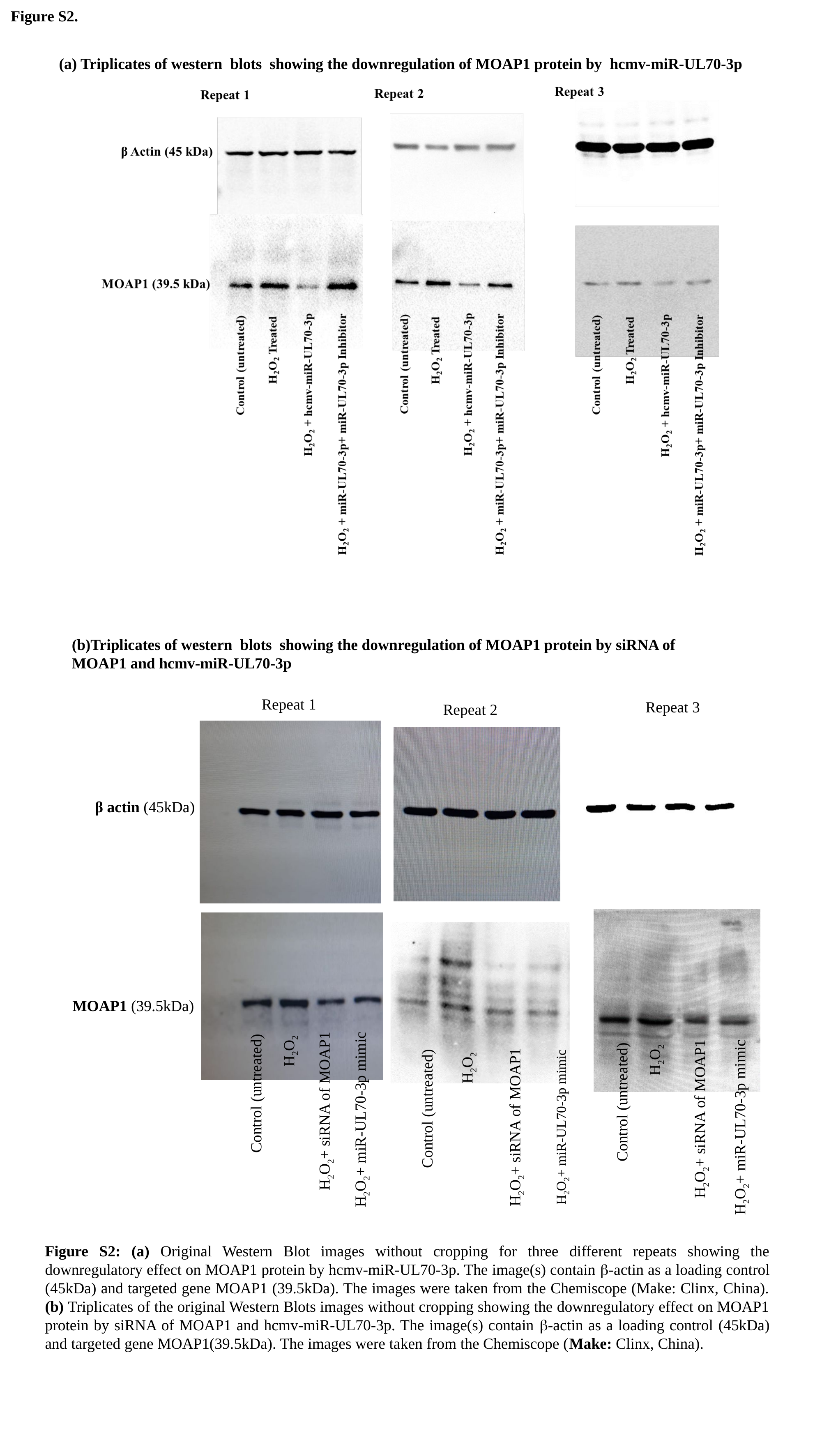

Figure S2.
(a) Triplicates of western blots showing the downregulation of MOAP1 protein by hcmv-miR-UL70-3p
(b)Triplicates of western blots showing the downregulation of MOAP1 protein by siRNA of MOAP1 and hcmv-miR-UL70-3p
Repeat 1
Repeat 3
Repeat 2
β actin (45kDa)
MOAP1 (39.5kDa)
H2O2
H2O2
H2O2
Control (untreated)
Control (untreated)
Control (untreated)
H2O2+ siRNA of MOAP1
H2O2+ siRNA of MOAP1
H2O2+ miR-UL70-3p mimic
H2O2+ siRNA of MOAP1
H2O2+ miR-UL70-3p mimic
H2O2+ miR-UL70-3p mimic
Figure S2: (a) Original Western Blot images without cropping for three different repeats showing the downregulatory effect on MOAP1 protein by hcmv-miR-UL70-3p. The image(s) contain β-actin as a loading control (45kDa) and targeted gene MOAP1 (39.5kDa). The images were taken from the Chemiscope (Make: Clinx, China). (b) Triplicates of the original Western Blots images without cropping showing the downregulatory effect on MOAP1 protein by siRNA of MOAP1 and hcmv-miR-UL70-3p. The image(s) contain β-actin as a loading control (45kDa) and targeted gene MOAP1(39.5kDa). The images were taken from the Chemiscope (Make: Clinx, China).
