# Supplementary material for: Human Cytomegalovirus miR-UL70-3p Downregulates the H2O2-Induced Apoptosis by Targeting the Modulator of Apoptosis-1 (MOAP1)
Source: Int J Mol Sci. 2021 Dec 21;23(1):18. doi: 10.3390/ijms23010018 (PMC8744590; doi:10.3390/ijms23010018)
Supplement: Supplementary file 1 [file ijms-23-00018-s001.zip › Figure S3.pptx]

## Slide 1
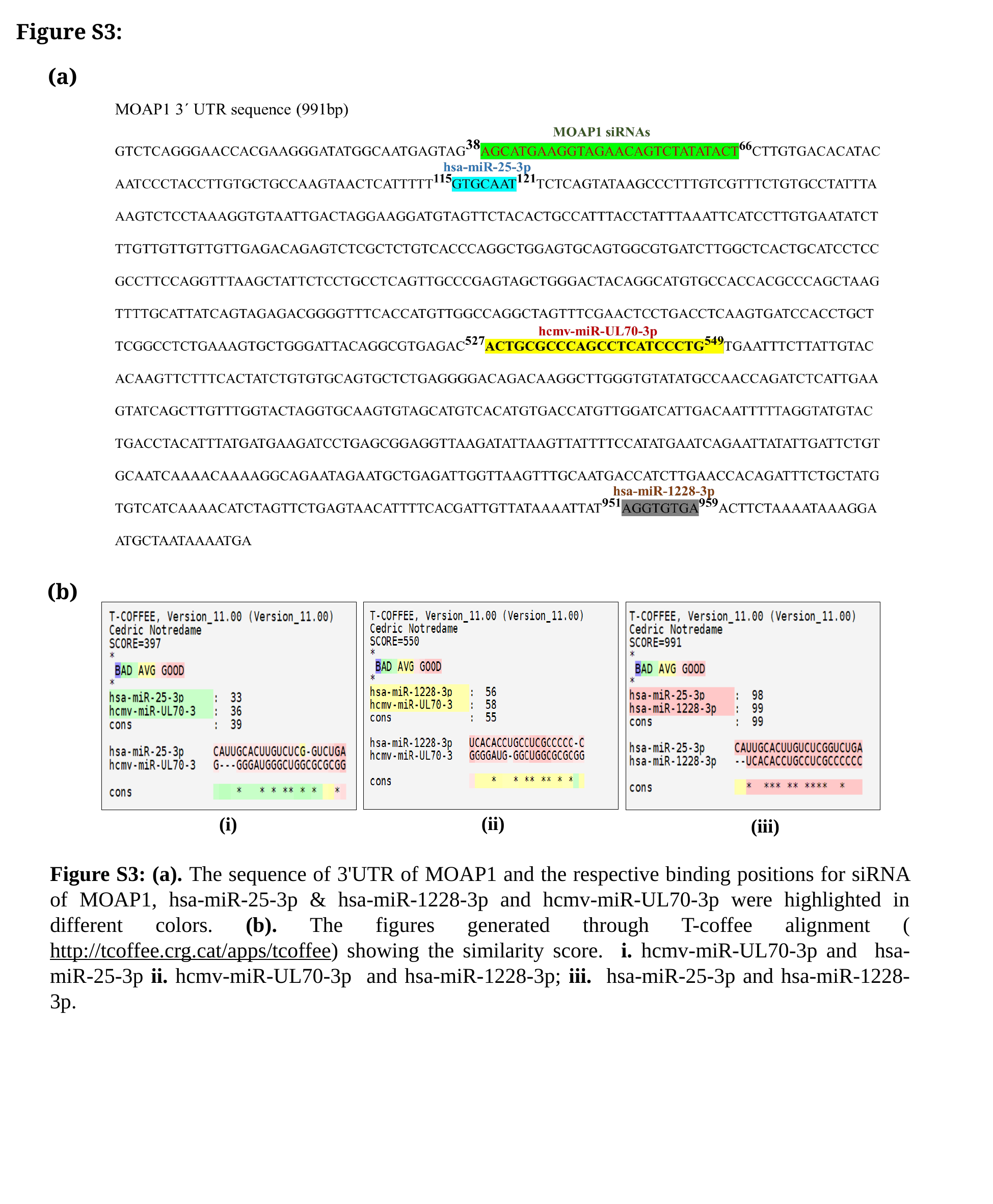

Figure S3:
(a)
(b)
(ii)
(i)
(iii)
Figure S3: (a). The sequence of 3'UTR of MOAP1 and the respective binding positions for siRNA of MOAP1, hsa-miR-25-3p & hsa-miR-1228-3p and hcmv-miR-UL70-3p were highlighted in different colors. (b). The figures generated through T-coffee alignment (http://tcoffee.crg.cat/apps/tcoffee) showing the similarity score. i. hcmv-miR-UL70-3p and hsa-miR-25-3p ii. hcmv-miR-UL70-3p and hsa-miR-1228-3p; iii. hsa-miR-25-3p and hsa-miR-1228-3p.
